# Supplementary material for: A hybrid single quantum dot coupled cavity on a CMOS-compatible SiC photonic chip for Purcell-enhanced deterministic single-photon emission
Source: Light Sci Appl. 2025 Feb 14;14:86. doi: 10.1038/s41377-024-01676-y (PMC11828937; doi:10.1038/s41377-024-01676-y)
Supplement: Supplementary file 1 — Supplementary information for: A hybrid single quantum dot coupled cavity on a CMOS-compatible SiC photonic chip for Purcell-enhanced deterministic single-photon emission [file 41377_2024_1676_MOESM1_ESM.docx]

Supplementary information for:

**A hybrid single quantum dot coupled cavity on a CMOS-compatible SiC photonic chip for Purcell-enhanced deterministic single-photon emission**

**Yifan Zhu,^1,2,*^ Runze Liu,^3,*^ Ailun Yi,^1,2,*^ Xudong Wang,^1,2^ Yuanhao Qin,^1,2^ Zihao Zhao,^1,2^ Junyi Zhao,^4,5^ Bowen Chen,^1,2^ Xiuqi Zhang,^1,2^ Sannian Song,^1,2^ Yongheng Huo,^4,5,†^ Xin Ou,^1,2,‡^ and Jiaxiang Zhang^1,2,§^**

*^1^State Key Laboratory of Materials for Integrated Circuits, Shanghai Institute of Microsystem and Information Technology, Chinese Academy of Sciences, 865 Changning Road, Shanghai, 200050, China*

*^2^Center of Materials Science and Optoelectronics Engineering, University of Chinese Academy of Sciences, Beijing, 100049, China*

*^3^Department of Physics, The Chinese University of Hong Kong, Shatin, New Terrotories, Hong Kong, 999077, China*

*^4^Hefei National Research Center for Physical Sciences at the Microscale and School of physical Sciences, University of Science and Technology of China, Hefei, 230026, China*

*^5^Shanghai Research Center for Quantum Science and CAS Center for Excellence in Quantum Information and Quantum Physics, University of Science and Technology of China, Shanghai, 201315, China*

*^*^These authors contributed equally to this work.*

*^†, ‡, §^Corresponding authors:* [*yongheng@ustc.edu.cn*](mailto:yongheng@ustc.edu.cn)*,* [*ouxin@mail.sim.ac.cn*](mailto:ouxin@mail.sim.ac.cn)*,* [*jiaxiang.zhang@mail.sim.ac.cn*](mailto:jiaxiang.zhang@mail.sim.ac.cn)*.*

**Supplemental Note 1: Device fabrication process**

Our quantum dots (QDs) sample was grown by molecular beam epitaxy and they were embedded within a 180 nm thick GaAs nanomembrane grown on a 200 nm thick Al_0.8_Ga_0.2_As sacrificial layer. Electron-beam lithography was then used to pattern tapered waveguide structures, followed by a dry etching using inductively coupled plasma (ICP) etching on the GaAs layer. Thereafter, the patterned GaAs sample was immersed in diluted hydrofluoricacid solution in order to selectively remove the sacrificial layer. With these nano-fabrications, free-standing GaAs nanophotonic waveguides were obtained, as shown in supplementary Fig. 1(i-iii). In parallel with the GaAs waveguides processing, a 4-inch wafer-scale thin-film 4H-SiC on Si/SiO_2_ substrate, that is, 4H-SiCOI, was prepared by using ion slicing and direct wafer bonding techniques (see more fabrication details in Ref.^1,2^). To fabricate photonic circuits, the wafer was then cut in small dies with a size of 1×1 cm^2^. A 100 kV electron-beam lithography and ICP-RIE (ULVAC, NE-550H) were subsequently used to fabricate photonic structures involving ring resonators and waveguides on the material platform. The thin 4H-SiC film was etched by 150 nm to form ridge waveguides. The width of the 4H-SiC waveguide was chosen to be (∼800 nm), which was slightly wider than that of GaAs tapered waveguide. This design ensures a single-mode operation of the waveguide at QDs emission wavelengths (∼910 nm). In the meantime it provides sufficient space to accommodate the GaAs tapered waveguide to be transferred. With the separately prepared GaAs waveguides and 4H-SiC photonic chip, we further adopted a deterministic transfer-printing technique to fabricate the hybrid GaAs/4H-SiC ring cavity^3-5^. As illustrated in supplementary Fig. 1ii∼Vi, the freestanding GaAs tapered waveguides were selectively picked up by a transparent Polydimethylsiloxane (PDMS) stamp. They were aligned relative to the 4H-SiC waveguides under a high-resolution microscope and then brought together until they contacted each other. Subsequently a tiny force was applied to form a Van der Waals force at the interfaces. In the last step, we peeled off the stamp very slowly to completely release the tapered GaAs waveguides.


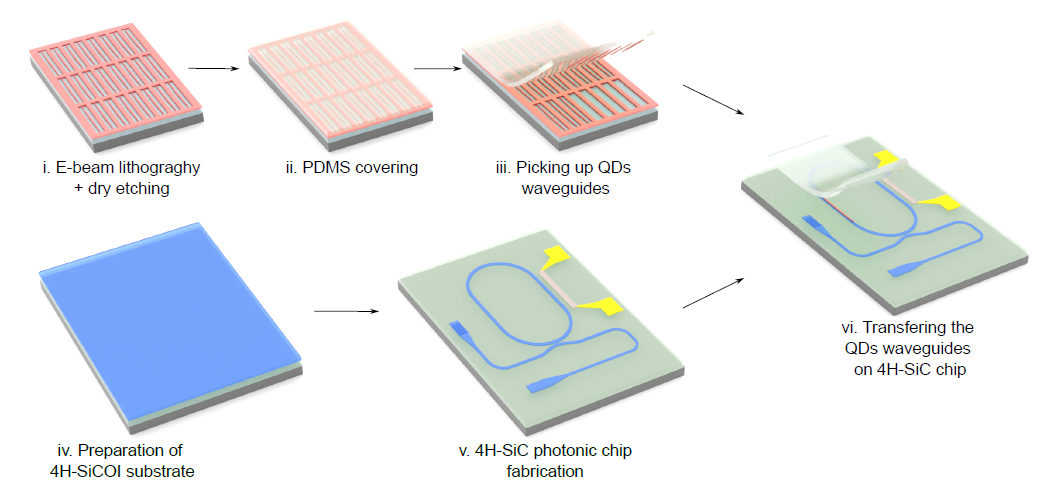


**Figure S1.** Detailed fabrication process of GaAs nanophotonic waveguides and 4H-SiC photonic chip, as well as the transfer-printing technique for fabricating GaAs/4H-SiC hybrid cavity.

**Supplemental Note 2: Coupling loss of the Mode transformer**

Fig. S2a shows a sketch of the mode transformer consisted of a tapered GaAs waveguide bounded onto the underlying 4H-SiC waveguide. These two waveguides are designed to preferentially support fundamental Transverse-Electrical like (TE-like) modes at *λ* ~ 910 nm. In the region (i), electric field of propagating wave is mainly confined inside the upper GaAs waveguide due to the high refractive index contrast between GaAs (*n*_GaAs_∼3.45) and 4H-SiC (*n*_4H−SiC_∼2.6) (see Fig. S2c). As GaAs waveguide tapers down from 400 nm to 80 nm, the fundamental TE-like mode evanescently couple into the underlying 4H-SiC waveguide, as shown by the bottom image in Fig. S2b and Fig. S2d. A near unity (∼98.3%) mode coupling efficiency at QDs emission of about 910 nm can be achieved for a 10 μm long taper (Fig. S2b).


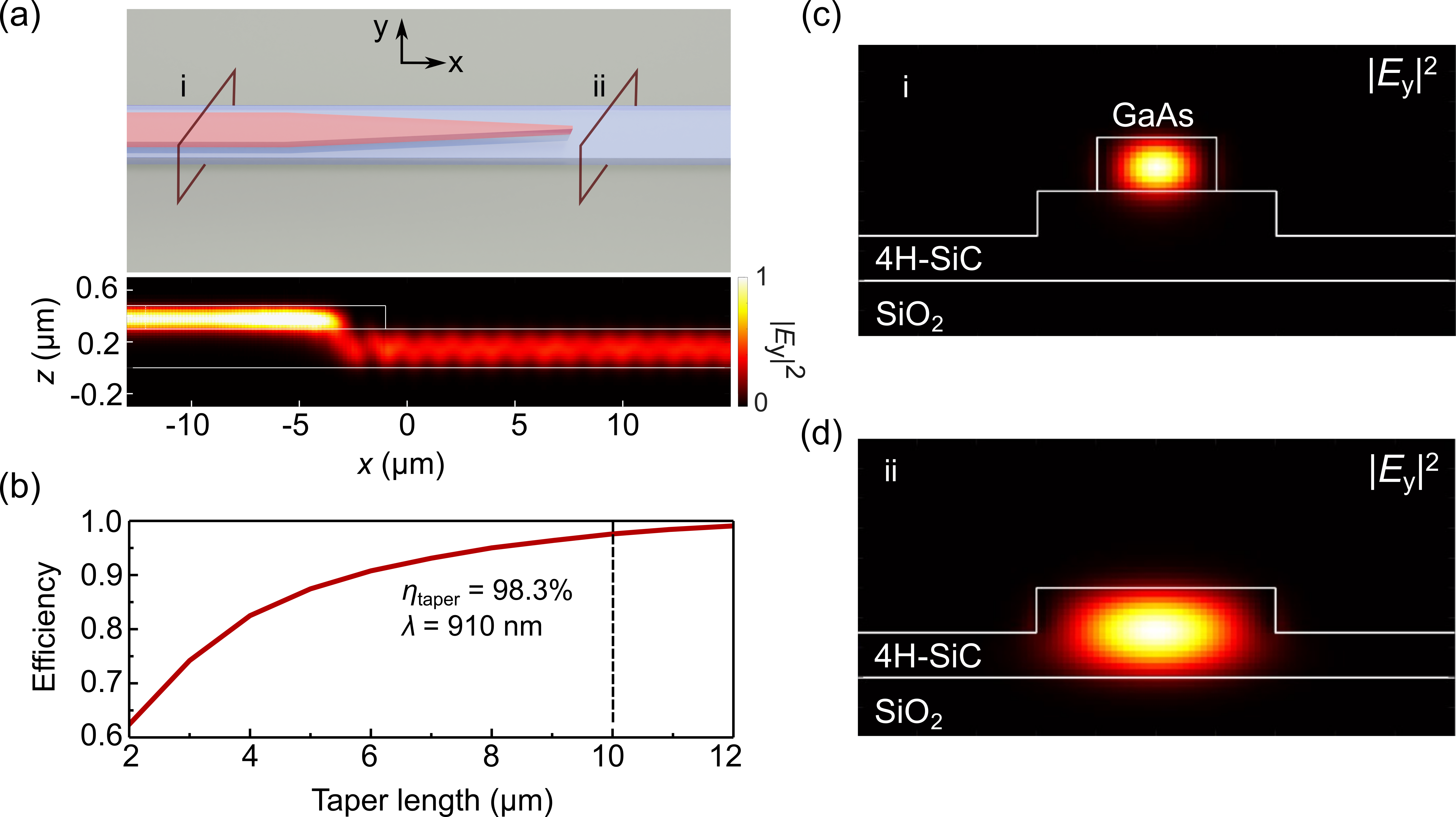


**Figure S2.** (a) A sketch of the mode transformer which consists of a tapered GaAs waveguide bonded onto the surface of 4H-SiC waveguide. The bottom panel shows the evanescent coupling of the fundamental TE-like mode through the top GaAs waveguide to the underlying 4H-SiC waveguide. (b) Simulated coupling efficiency of the mode transformer as a function of taper length. (c), (d) Mode filed profile at different positions in the mode transformer.

As for *α*_taper_ in the main text, it depends on geometric structure of the hybrid GaAs/SiC tapered waveguide, we can perform numerical simulations to optimize the geometric parameters in order to achieve an optimal mode coupling efficiency. Fig. S3a and S3b plot the simulated coupling efficiency *α*_taper_ as a function of the tip width and the length of the GaAs taper, respectively. For quantum dot emission at 910 nm, an optimal *η*_taper_ = 99.8% is found when the tip width is set to 100 nm and taper length set to 10 μm. Consequently, *α*_taper_ is calculated to be 0.869 dB mm^-1^. This value is one-order of magnitude lower than the value reported in the original manuscript (7.45 dB mm^-1^). With the optimal losses, the *Q* factor can be improved by one-order of magnitude and therefore the Purcell factor can be enhanced to the same extent. Fig. S3c shows the wavelength-dependent *α*_taper_ for different tip widths. Optimal results can be obtained for tip width at about 100 nm.


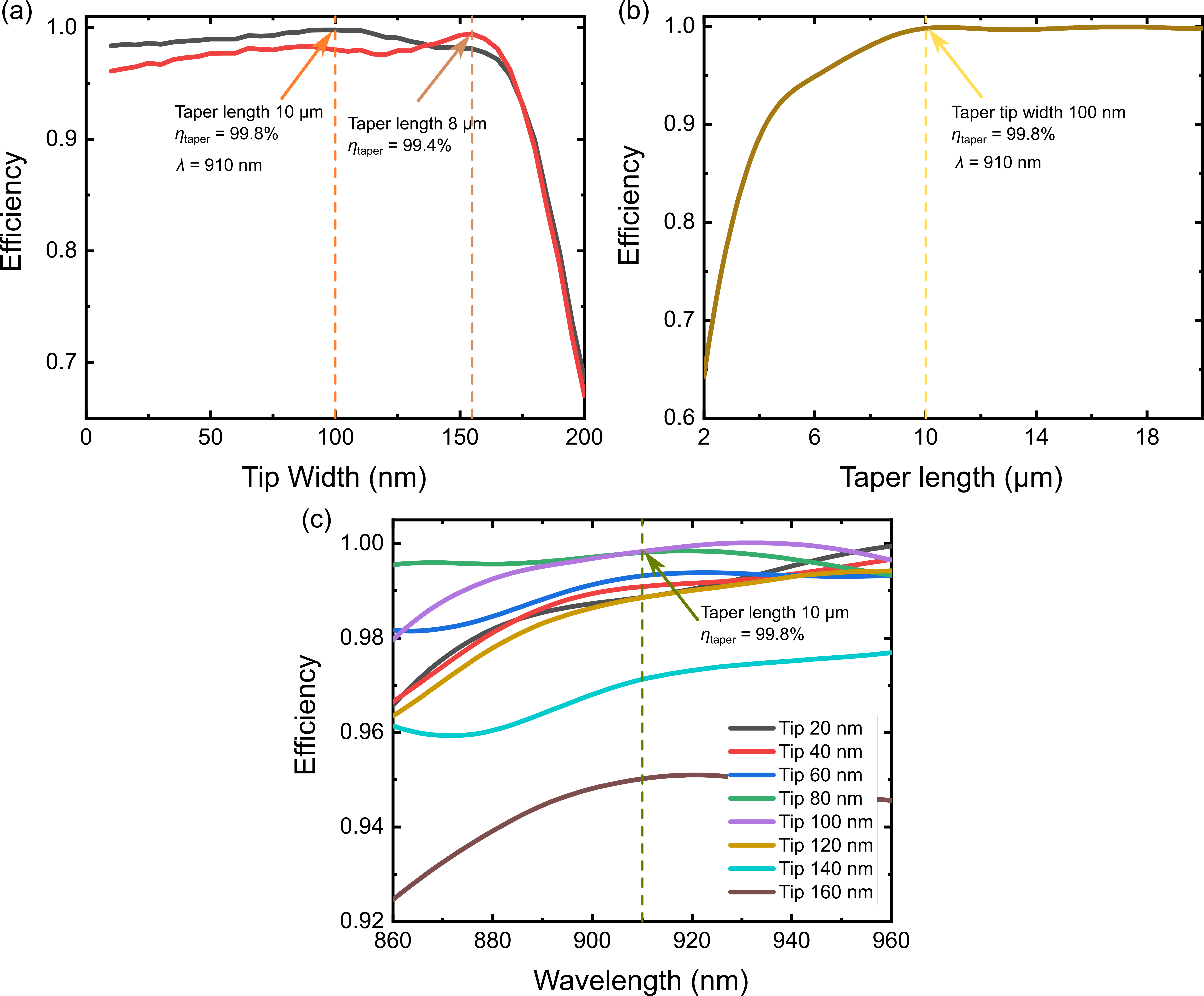


**Figure S3.** (a) Simulated coupling efficiency *α*_taper_ as a function of the tip width for different lengths of the taper. (b) Simulated coupling efficiency *α*_taper_ of nm as a function of the taper length for a fixed tip width of 100 nm. (c) The wavelength-dependent coupling efficiency of the mode transformer for different tip widths when the taper length is fixed to 10 μm.

**Supplemental Note 3: Experimental setup**

For optical measurements, the hybrid quantum photonic chip was placed in a closed-cycle (Montana, Fusion F2) and all measurements were carried out at about 6 K. Objective lens (50×, N.A. = 0.65, Mitutoyo) was placed inside the cryostat to improve the system stability. A 3-axis nanopositioner (Nano Precision (Shanghai) Inc.) was used to move the sample with a nanometer precision at cryogenic temperature. A home-built micro-photoluminescence (μ-PL) setup was used to characterize the hybrid photonic chip. The excitation and collection fibers (single mode fiber, SMF) as well as their associated optics were assembled within a 30 mm cage system and mounted on the top of the cryostat. Optical excitation was performed off-resonantly by a continuous wave 532 nm laser, which was coupled into the μ-PL setup by the objective. The PL signal was coupled into a SMF. For optical measurements, photoluminescence from either the QDs source position or the grating couplers was collected by the same objective and then was delivered to the collection fiber. The photoluminescence of QDs were selectively filtered by using a home-built grating filter (resolution ∼0.2 nm), and then directed to a 750 mm high-resolution spectrometer equipped with a charge-coupled device (CCD). Time-resolved photoluminescence, as well as photon correlation, were measured by a Hanbury Brown and Twiss (HBT) setup which consists of a non-polarizing 50:50 beam splitter, two identical superconducting nanowire single-photon detectors, a single-photon counting module (Picoharp 300) and other polarization optical components.


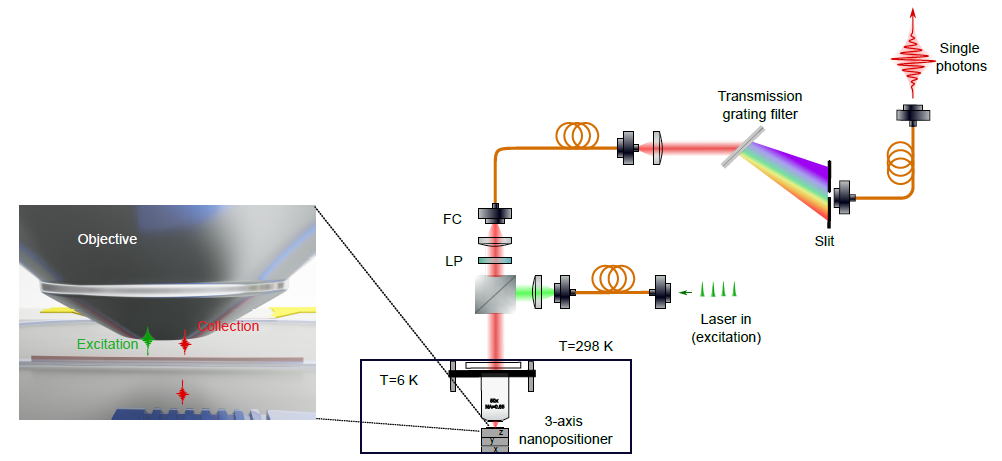


**Figure S4.** Optical experimental setup. The left image illustrates the light excitation and collection scheme in our experiment.

**Supplemental Note 4: Analysis of the limiting factors for optimal Purcell factor of the hybrid cavity**

For our hybrid QDs-coupled cavity, there exists a discrepancy between the theoretically simulated Purcell factor and the experimentally achieved value. In fact, this discrepancy can be ascribed to the random distribution of the QD position and its dipole orientation with respect to the nanophotonic waveguide. To evaluate their impacts on the Purcell factor, we have performed theoretical simulations and the results are shown in Fig. S5. As the quantum dot moves from the waveguide center to the edge, the Purcell factor drops dramatically from about 6 to almost zero. Same change takes place for the variations of the quantum dot orientation. In fact, these results can be interpreted as a consequence of the mode overlap change between the quantum dot dipole and the waveguide mode field. Since the waveguide is designed to preferentially support the fundamental TE-like mode where the mode field maximum locates at the waveguide center and the mode polarization is perpendicular to the waveguide. Deviations in both the dot position and polarization will lead to a decrease in the mode overlap integral. Therefore, in order to achieve the optimal Purcell factor, further works can be adopted to optimize the quantum dot position and its dipole polarization. One of the promising ways is to precisely position quantum dots in the waveguide via a wide-filed imaging technique^6^.


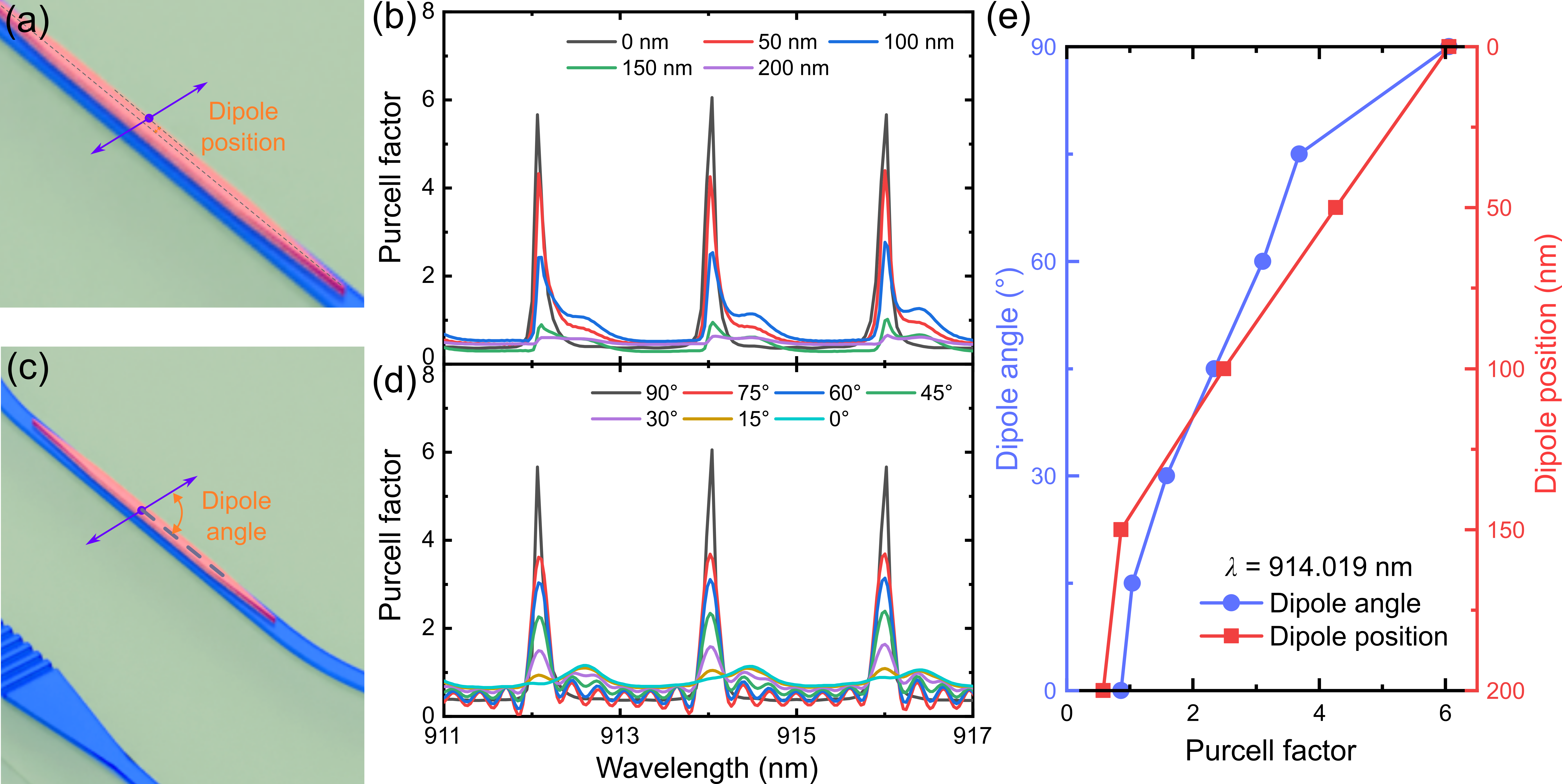


**Figure S5.** (a) Sketch of QD related dipole position inside the upper GaAs waveguide and it can be laterally moved from the center to the edge. (b) Position-dependent Purcell factor in the GaAs waveguide. (c) Sketch of QD related dipole orientation with respect to the GaAs waveguide. (d) Angle-dependent Purcell factor of the QDs in GaAs waveguide. (e) Purcell factor vs dipole position and dipole angle for a fixed wavelength at 914 nm.

**Supplemental Note 5: Comparison with the state-of-the-art devices on QDs-based hybrid quantum photonic platforms**

We have summarized all hybrid integrated quantum photonics with self-assembled quantum dots, as shown in Supplementary Table 1. In the comparison, we can find that our device outperforms these state-of-the-art results in terms of wider tuning range (~ 4 nm), local tuning capability, highest Purcell factor and purity of deterministic single-photon emission. All these metrics, despite not optimal as compared to the off-chip device, make our chip particularly appealing for the development of scalable on-chip quantum light sources.

**Table S1:** The state-of-the-art works for hybrid integrated quantum photonics based on QDs.

| Photonic structures | | Tuning method | Tuning range | Local tuning | *g*^(2)^(0) | Purcell factor |
| --- | --- | --- | --- | --- | --- | --- |
| GaAs micro-ring on Si_3_N_4_ waveguide^7^ | 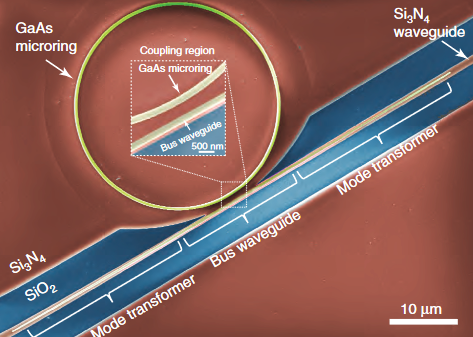 | Nitrogen gas-tuning mechanism | 1.1 nm | No | 0.07 ±0.01 | 4 |
| GaAs nanobeam on Si waveguide^8^ | 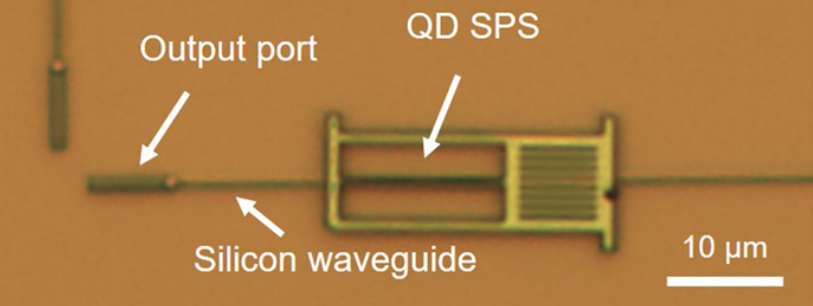 | Temperature | >2 nm | No | 0.30 | ≈2 |
| InP QDs on Si waveguide^9^ | 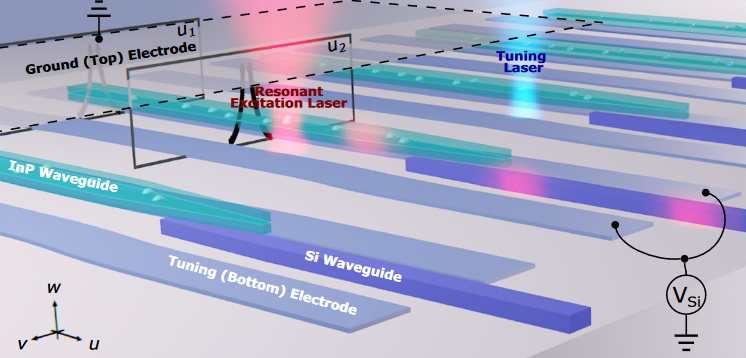 | Electric field | <0.5 nm | Yes | 0.12 ±0.03 | / |
| GaAs nanobeam on GaAs waveguide^10^ | 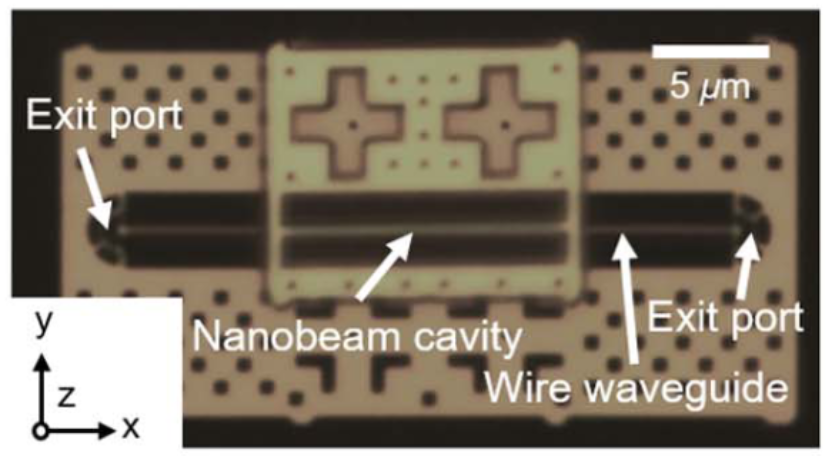 | Temperature | >1.1 nm | No | 0.23 | 3.9 |
| GaAs nanobeam on Si waveguide^11^ | 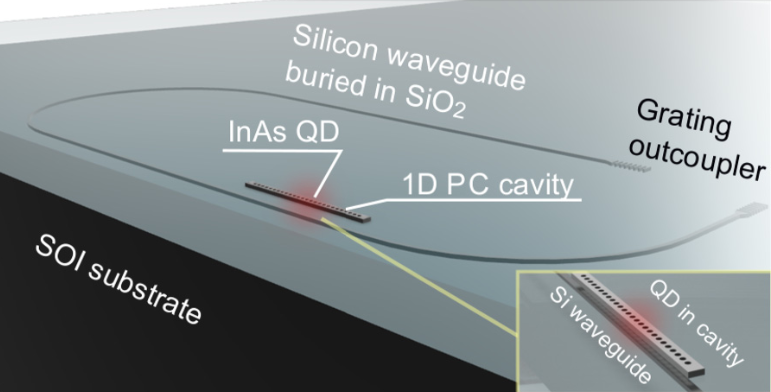 | Temperature | 0.8 nm | No | / | / |
| GaAs on SiN waveguide ^12^ | 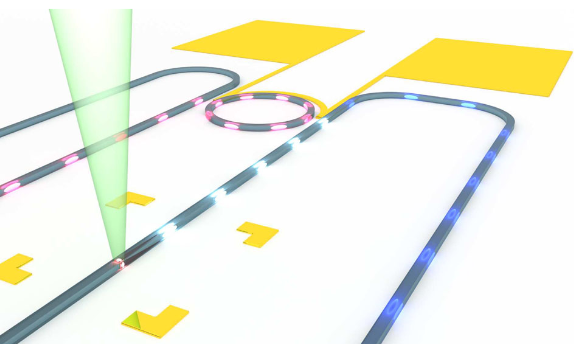 | Micro-heater | >1.2 nm | Yes | 0.13 ±0.04 | / |
| This work | 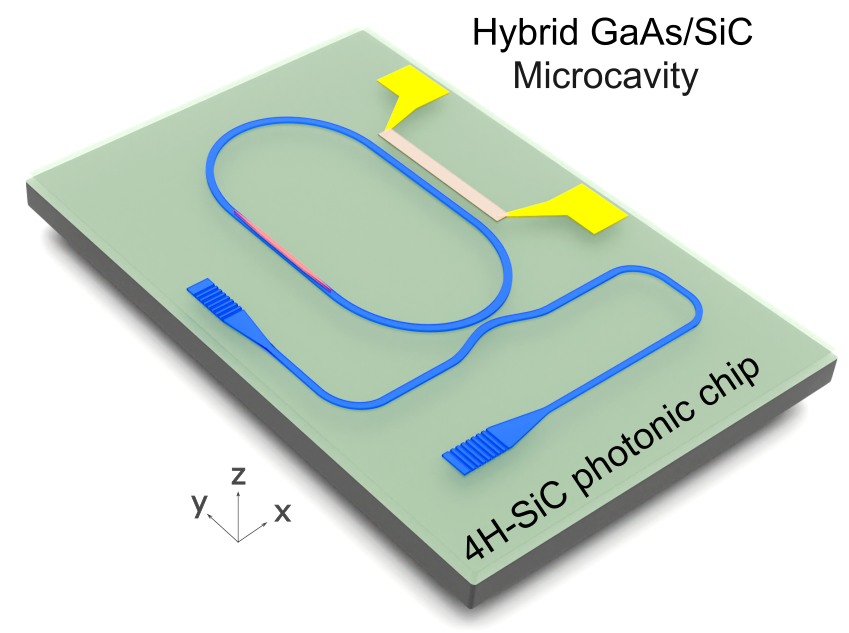 | Micro-heater | ~4 nm | Yes | 0.008 ±0.004 | 4.9 |

**References**

1 Yi, A. *et al.* Wafer-scale 4H-silicon carbide-on-insulator (4H–SiCOI) platform for nonlinear integrated optical devices. *Opt. Mater.* **107**, 109990, (2020).

2 Zhu, Y. *et al.* Hybrid Integration of Deterministic Quantum Dot-Based Single-Photon Sources with CMOS-Compatible Silicon Carbide Photonics. *Laser Photonics Rev.* **16**, 2200172, (2022).

3 Elshaari, A. W., Pernice, W., Srinivasan, K., Benson, O. & Zwiller, V. Hybrid integrated quantum photonic circuits. *Nat. Photon.* **14**, 285-298, (2020).

4 Kim, J.-H., Aghaeimeibodi, S., Carolan, J., Englund, D. & Waks, E. Hybrid integration methods for on-chip quantum photonics. *Optica* **7**, 291-308, (2020).

5 Jin, T. *et al.* Generation of Polarization-Entangled Photons from Self-Assembled Quantum Dots in a Hybrid Quantum Photonic Chip. *Nano Lett.* **22**, 586-593, (2022).

6 Liu, J. *et al.* A solid-state source of strongly entangled photon pairs with high brightness and indistinguishability. *Nature nanotechnology* **14**, 586–593-586–593, (2019).

7 Davanco, M. *et al.* Heterogeneous integration for on-chip quantum photonic circuits with single quantum dot devices. *Nature Communications* **8**, 889, (2017).

8 Katsumi, R. *et al.* Quantum-dot single-photon source on a CMOS silicon photonic chip integrated using transfer printing. *APL Photonics* **4**, 036105, (2019).

9 Larocque, H. *et al.* Tunable quantum emitters on large-scale foundry silicon photonics. *Nature Communications* **15**, 5781, (2024).

10 Katsumi, R., Ota, Y., Kakuda, M., Iwamoto, S. & Arakawa, Y. Transfer-printed single-photon sources coupled to wire waveguides. *Optica* **5**, 691, (2018).

11 Osada, A. *et al.* Strongly coupled single-quantum-dot–cavity system integrated on a CMOS-processed silicon photonic chip. *Physical Review Applied* **11**, 024071-024071, (2019).

12 Elshaari, A. W. *et al.* On-chip single photon filtering and multiplexing in hybrid quantum photonic circuits. *Nature communications* **8**, 379-379, (2017).
